# Supplementary material for: The Potential of Fluralaner as a Bait Toxicant to Control Pest Yellowjackets in California
Source: Insects. 2023 Mar 23;14(4):311. doi: 10.3390/insects14040311 (PMC10143787; doi:10.3390/insects14040311)
Supplement: Supplementary file 1 [file insects-14-00311-s001.zip › insects-2261186-supplementary.pdf]

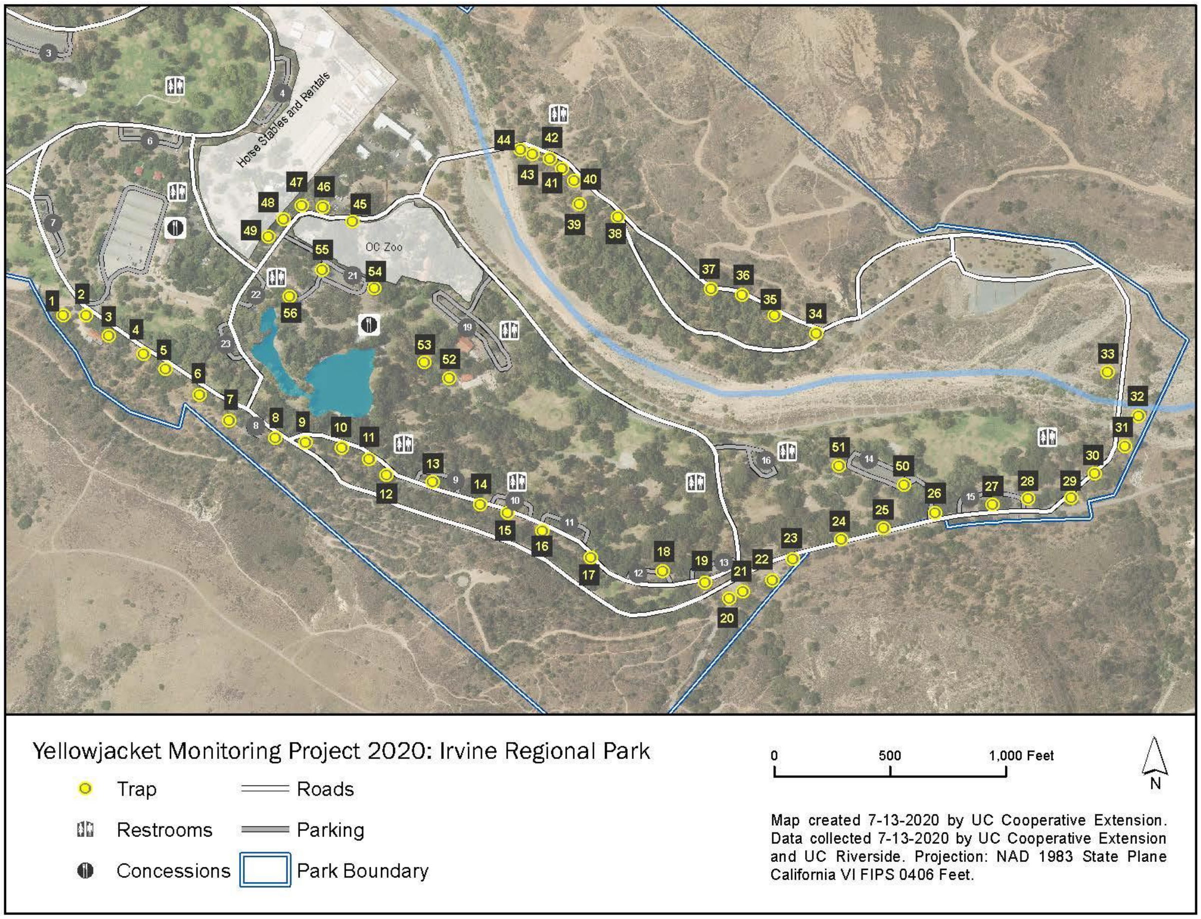

Figure S1. Location of monitoring traps at IRP. Choice arenas were deployed at monitoring sites 38, 43, and 50.

att

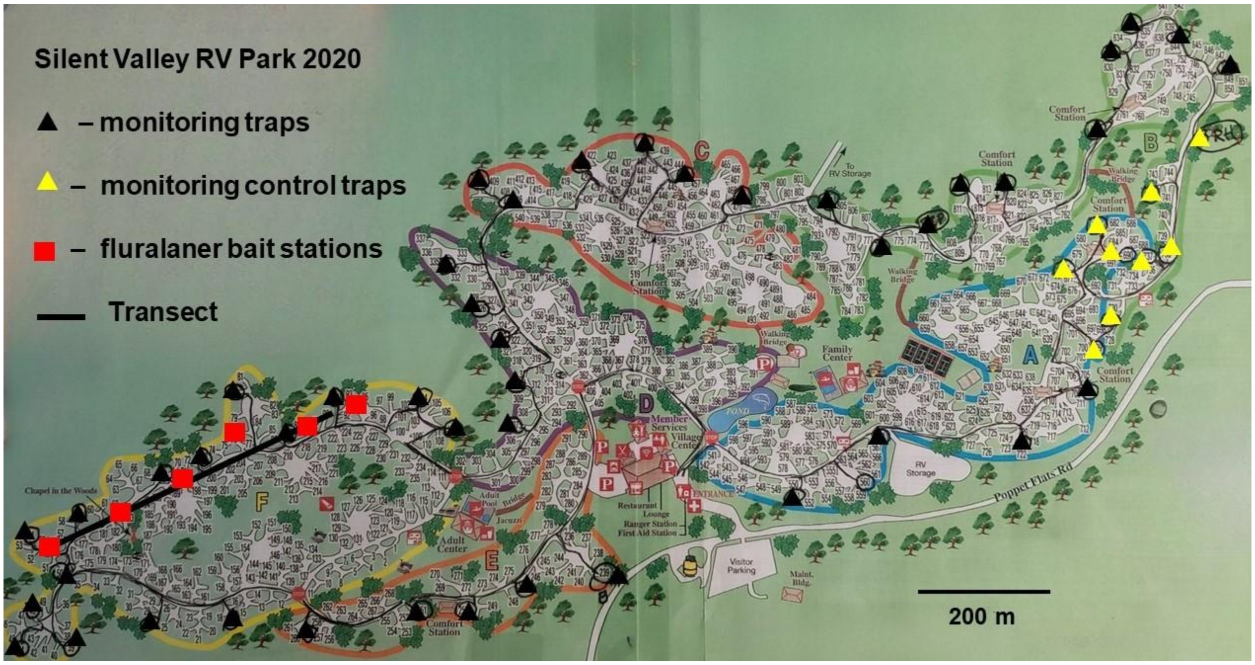

Figure S2. Location of monitoring traps and baiting stations at SV.
